# Risk-adapted trajectory selection for ultrasound-guided thyroid fine-needle aspiration biopsy: a multicenter study of in-plane and out-of-plane approaches

**DOI:** 10.3389/fendo.2026.1908297

**Published:** 2026-07-17

**Authors:** Xiubin Tang, Wenjin Lin, Youhai Wu, Ming Chen, Xiao Yang

**Affiliations:** 1 Department of Ultrasound, Zhangzhou Affiliated Hospital of Fujian Medical University, Zhangzhou, China; 2 Department of Ultrasound, The Union Hospital Affiliated to Fujian Medical University, Fuzhou, China; 3 Department of Ultrasound, The Second Affiliated Hospital of Fujian Medical University, Quanzhou, China; 4 Department of Ultrasound, Chinese Academy of Medical Sciences and Peking Union Medical College, Beijing, China

**Keywords:** diagnostic adequacy, fine-needle aspiration biopsy, needle-tip visualization, thyroid nodule, ultrasound guidance

## Abstract

**Background:**

Ultrasound-guided fine-needle aspiration biopsy (FNAB) is widely used for thyroid nodule evaluation, but nondiagnostic cytology can lead to repeat procedures and delayed management. Because needle approach is influenced by nodule size, anatomical risk, access route, and needle-tip visibility, simple in-plane versus out-of-plane comparisons may not fully reflect clinical decision-making. This study evaluated the association between needle approach and diagnostic adequacy and explored whether the findings could inform a preliminary, risk-adapted trajectory-selection concept.

**Methods:**

This multicenter retrospective cohort study included 506 thyroid nodules that underwent clinically indicated ultrasound-guided FNAB between September 2024 and December 2025 at three tertiary hospitals. One center formed the primary cohort, and two independent centers formed the external validation cohort. Diagnostic adequacy was defined as Bethesda category II-VI cytology, and Bethesda category I cytology was retained in the analysis as a nondiagnostic outcome rather than excluded. Multivariable logistic regression was used to evaluate the association between needle approach and diagnostic adequacy, with adjustment for nodule size category, complex anatomical location, operator experience, and center source in pooled analyses. Stabilized inverse probability of treatment weighting was performed as a sensitivity analysis.

**Results:**

Among 506 nodules, 289 were sampled using the in-plane approach and 217 using the out-of-plane approach. Diagnostic adequacy was higher with the in-plane approach than with the out-of-plane approach (98.3% vs. 85.7%; *P* < 0.001). The adjusted association remained significant in the primary cohort (adjusted odds ratio [OR], 9.39; 95% confidence interval [CI], 2.37–37.18), external validation cohort (adjusted OR, 14.51; 95% CI, 2.82–74.74), and pooled center-adjusted model (adjusted OR, 12.10; 95% CI, 4.28–34.20). Weighted sensitivity analysis yielded similar results. Satisfactory needle-tip visualization was more frequent with the in-plane approach (97.2% vs. 63.6%; *P* < 0.001), whereas immediate hematoma did not differ significantly between approaches (6.2% vs. 9.2%; *P* = 0.234).

**Conclusion:**

The in-plane approach was associated with higher diagnostic adequacy without a statistically significant increase in immediate hematoma. These findings provide preliminary, hypothesis-generating multicenter evidence that may inform risk-adapted trajectory selection for ultrasound-guided thyroid FNAB.

## Introduction

Ultrasound-guided fine-needle aspiration biopsy (FNAB) is an established method for evaluating thyroid nodules and is incorporated into contemporary thyroid nodule management and cytopathology reporting frameworks (1–3). Practical technical reviews also describe ultrasound-guided FNAB as a standard image-guided thyroid biopsy procedure (4). Its diagnostic value depends not only on accurate needle access to the target lesion but also on the acquisition of material adequate for cytological interpretation. In the Bethesda reporting system, Bethesda I denotes nondiagnostic cytology, which may lead to repeat biopsy, alternative tissue sampling, and delays in clinical decision-making (1). Diagnostic adequacy therefore remains a clinically relevant endpoint in thyroid FNAB, particularly because sampling success is influenced by both lesion-related characteristics and procedural factors (5, 6).

Both in-plane and out-of-plane approaches are routinely used for ultrasound-guided thyroid FNAB, but they differ in needle visualization and technical execution. The in-plane approach permits continuous visualization of the needle trajectory within the ultrasound imaging plane, whereas the out-of-plane approach relies on identification of the needle tip as it intersects the imaging plane. Previous comparisons of long-axis/in-plane and short-axis/out-of-plane techniques suggest that needle approach may affect procedural performance (7, 8). Simulation-based assessments further indicate that these approaches differ in operator perception and technical control, with potential implications for needle-tip confirmation during target sampling (9). However, how these technical differences should inform individualized trajectory selection in thyroid FNAB remains insufficiently defined.

In clinical practice, needle approach is rarely determined by imaging-plane orientation alone. Target size, skin-to-nodule distance, proximity to the trachea or major cervical vessels, acoustic window availability, operator familiarity, and the feasibility of reliable needle-tip confirmation all contribute to trajectory planning. In this manuscript, risk-adapted trajectory selection refers to individualized selection of an in-plane or out-of-plane needle path according to nodule size, anatomical relationship to critical structures, safe access route, and expected needle-tip visibility; it does not refer to a validated decision algorithm or to operator preference alone. Nodule size is particularly relevant because maximum diameter may influence FNAB outcomes and sampling adequacy (10). More broadly, contemporary thyroid nodule management increasingly favors risk-adapted decision-making rather than uniform procedural strategies (2). Thus, a simple comparison between in-plane and out-of-plane techniques may insufficiently capture the clinical reasoning that guides approach selection.

This multicenter retrospective cohort study examined the association between needle approach and diagnostic adequacy and explored whether the observed findings could inform a preliminary, risk-adapted trajectory-selection concept for thyroid FNAB. A primary cohort was used to assess this association, and two independent external validation centers were used to examine its directional consistency across clinical settings. Needle-tip visualization was analyzed as a procedural process variable to assess whether reliable tip confirmation provided procedural support for the observed association. Procedure-related hematoma was evaluated as the predefined safety endpoint, consistent with the need to characterize FNAB-related harms using objective and clearly documented outcomes (11). By moving beyond a fixed in-plane versus out-of-plane comparison, this study sought to provide preliminary, hypothesis-generating multicenter evidence for FNAB trajectory selection based on nodule size, anatomical risk, access safety, and expected needle-tip confirmation.

## Materials and methods

### Study design and cohorts

In this multicenter retrospective cohort study, consecutive thyroid nodules undergoing clinically indicated ultrasound-guided fine-needle aspiration biopsy (FNAB) between September 2024 and December 2025 were screened at three tertiary hospitals in Fujian Province, China. The primary cohort comprised 303 eligible nodules from Zhangzhou Affiliated Hospital of Fujian Medical University. This lead center initiated the standardized variable-extraction framework and was used to evaluate the association between needle approach and diagnostic adequacy. The independent external validation cohort comprised 203 eligible nodules with complete records available for analysis from the Union Hospital Affiliated to Fujian Medical University (Fuzhou, China; 113 nodules) and the Second Affiliated Hospital of Fujian Medical University (Quanzhou, China; 90 nodules). These two centers were analyzed as an external validation cohort to examine the directional consistency of the association across separate clinical settings. For the external validation cohort, consecutive eligible nodules were included when complete ultrasound, procedural, needle-approach, needle-tip visualization, and cytological records were available for centralized data analysis.

This study was conducted in accordance with the Declaration of Helsinki. Ethical approval was obtained from the Ethics Committee of the Zhangzhou Affiliated Hospital of Fujian Medical University (Approval No. 2025LWB215). Further details regarding external-center data authorization and the waiver of informed consent are provided in the Ethics statement.

### Eligibility criteria

**Figure 1:**
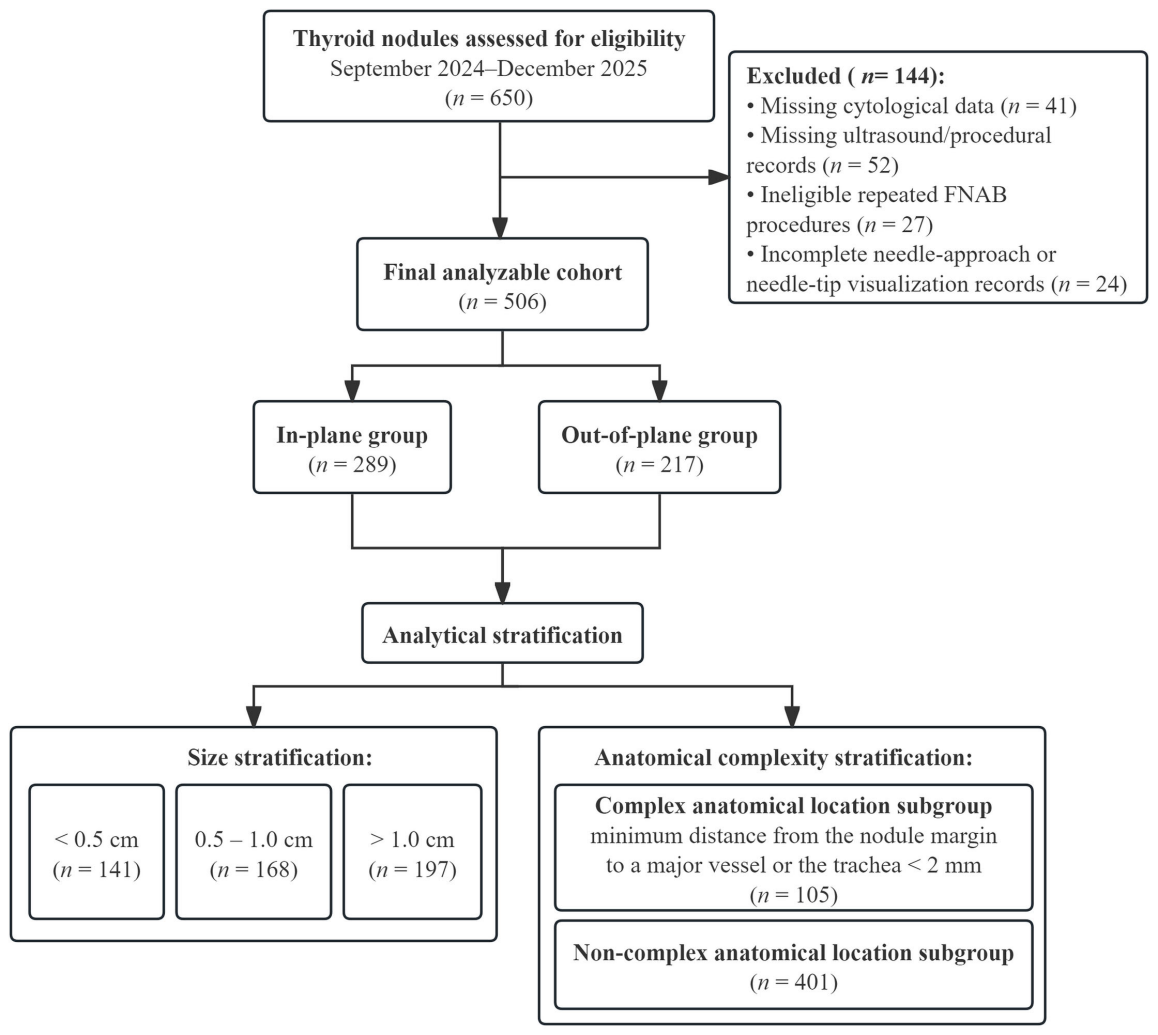
Study flow, needle-approach grouping, and analytical stratification. Consecutive thyroid nodules undergoing clinically indicated ultrasound-guided FNAB between September 2024 and December 2025 were screened. Of 650 screened nodules, 144 were excluded because of unavailable cytological data (n = 41), unavailable ultrasound or procedural records (n = 52), ineligible repeated FNAB procedures (n = 27), or incomplete needle-approach or needle-tip visualization records (n = 24), leaving 506 nodules in the final analyzable cohort. The 41 nodules with unavailable cytological data had no retrievable formal Bethesda cytology report and were distinct from Bethesda category I nondiagnostic cases, which were retained as nondiagnostic outcomes in the final analysis. The figure also shows needle-approach grouping and predefined stratification by nodule size and anatomical complexity. FNAB, fine-needle aspiration biopsy.

Consecutive thyroid nodules that underwent clinically indicated ultrasound-guided FNAB between September 2024 and December 2025 were screened for eligibility. Nodules were included if complete ultrasound, procedural, and cytological records were available. Inclusion required documentation of the needle approach and cytological classification according to the Bethesda System for Reporting Thyroid Cytopathology. Bethesda I cytology was not excluded from the final analyzable cohort; it was retained and coded as nondiagnostic cytology for the primary outcome analysis. When more than one thyroid nodule was present in the same patient, only the index nodule undergoing clinically indicated FNAB during the study period was included; therefore, each patient contributed only one nodule to the analysis. Of 650 screened nodules, 144 were excluded because cytological data were unavailable (n = 41), ultrasound or procedural records could not be retrieved (n = 52), the biopsy represented an ineligible repeated FNAB procedure (n = 27), or needle-approach or needle-tip visualization records were incomplete (n = 24). The 41 nodules with missing cytological data had no retrievable formal Bethesda cytology report and were therefore distinct from Bethesda I nondiagnostic cases, which remained in the outcome analysis. Because the specific downstream reasons for unavailable cytology could not be reliably reconstructed from the anonymized retrospective records, these cases were reported as a separate exclusion category rather than reclassified as nondiagnostic cytology. A complete-case analysis was performed, and no statistical imputation was applied because the primary exposure and primary outcome were required for cohort eligibility. The final analyzable cohort comprised 506 thyroid nodules. **Figure 1** shows the study flow, exclusion categories, needle-approach grouping, and predefined analytical stratification.

### Ultrasound-guided FNAB procedure

All FNAB procedures were performed under continuous real-time ultrasound guidance using high-frequency linear transducers. A standardized 23-gauge, 9-cm needle (PCN23/09; Gallini S.R.L., Mantova, Italy) was used for thyroid FNAB. A needle pass was defined as a single needle insertion into the target nodule for cytological sampling. In the included procedures from all three centers, each target nodule underwent two documented needle passes, and no additional passes were recorded. This reflected the standardized practice captured in the study dataset rather than a universal requirement for thyroid FNAB outside this retrospective cohort.

Needle approach was recorded as the primary exposure variable and classified as in-plane or out-of-plane. The in-plane approach was defined as advancement of the needle within the ultrasound imaging plane, allowing visualization of the needle trajectory during target sampling. The out-of-plane approach was defined as needle advancement perpendicular or oblique to the imaging plane, with needle-tip localization based on its intersection with the ultrasound plane. Rapid on-site evaluation (ROSE) was not routinely performed as part of the FNAB workflow during the study period, which may have influenced adequacy assessment and the decision not to perform additional passes during the recorded procedures.

### Variable definitions

Nodule size was categorized based on the maximum diameter on preprocedural ultrasound: < 0.5 cm, 0.5–1.0 cm, or > 1.0 cm. Complex anatomical location was defined as a nodule located within 2 mm of the trachea or a major cervical vessel. This predefined 2-mm cutoff was selected as a pragmatic imaging threshold for close adjacency to critical structures, where the available safety corridor is narrow and trajectory planning is expected to be more technically constrained. Skin-to-nodule distance was measured along the planned puncture trajectory from the skin surface to the nearest nodule margin.

Needle-tip visualization was retrospectively assessed based on archived FNAB ultrasound imaging materials, including dynamic video clips and static images, and was classified as satisfactory or unsatisfactory according to predefined criteria. Satisfactory needle-tip visualization was defined as clear confirmation of the needle tip within the target nodule during sampling. For both in-plane and out-of-plane procedures, dynamic video clips were preferentially reviewed when available to confirm needle-tip position during target sampling; static images were used as supplemental source materials when dynamic clips were unavailable or incomplete. The availability and quality of dynamic clips were not prospectively standardized across all procedures. Two independent ultrasound physicians with more than 5 years of thyroid interventional ultrasound experience reviewed the available imaging materials while blinded to cytological outcomes, which were determined from formal cytopathology reports. Disagreements were resolved by consensus, and interobserver agreement for needle-tip visualization classification was assessed using Cohen’s kappa in the available reviewed imaging materials. Image reviewers were blinded to cytological outcomes but not to needle approach because procedural features were visible on the ultrasound images. Needle-tip visualization was therefore analyzed as a descriptive procedural process variable rather than as a formally tested mediator.

Operator experience was categorized uniformly across centers. Senior operators were defined as attending-level or higher ultrasound physicians with at least 5 years of independent thyroid FNAB experience, and all other operators were categorized as non-senior. Operators at each center were trained in both in-plane and out-of-plane FNAB approaches. Operator-level information was summarized descriptively in **Supplementary Table 1** to characterize procedural practice patterns; however, these descriptors were not incorporated into the primary multivariable model because of the limited number of nondiagnostic events and the risk of model overfitting.

**Figure 2:**
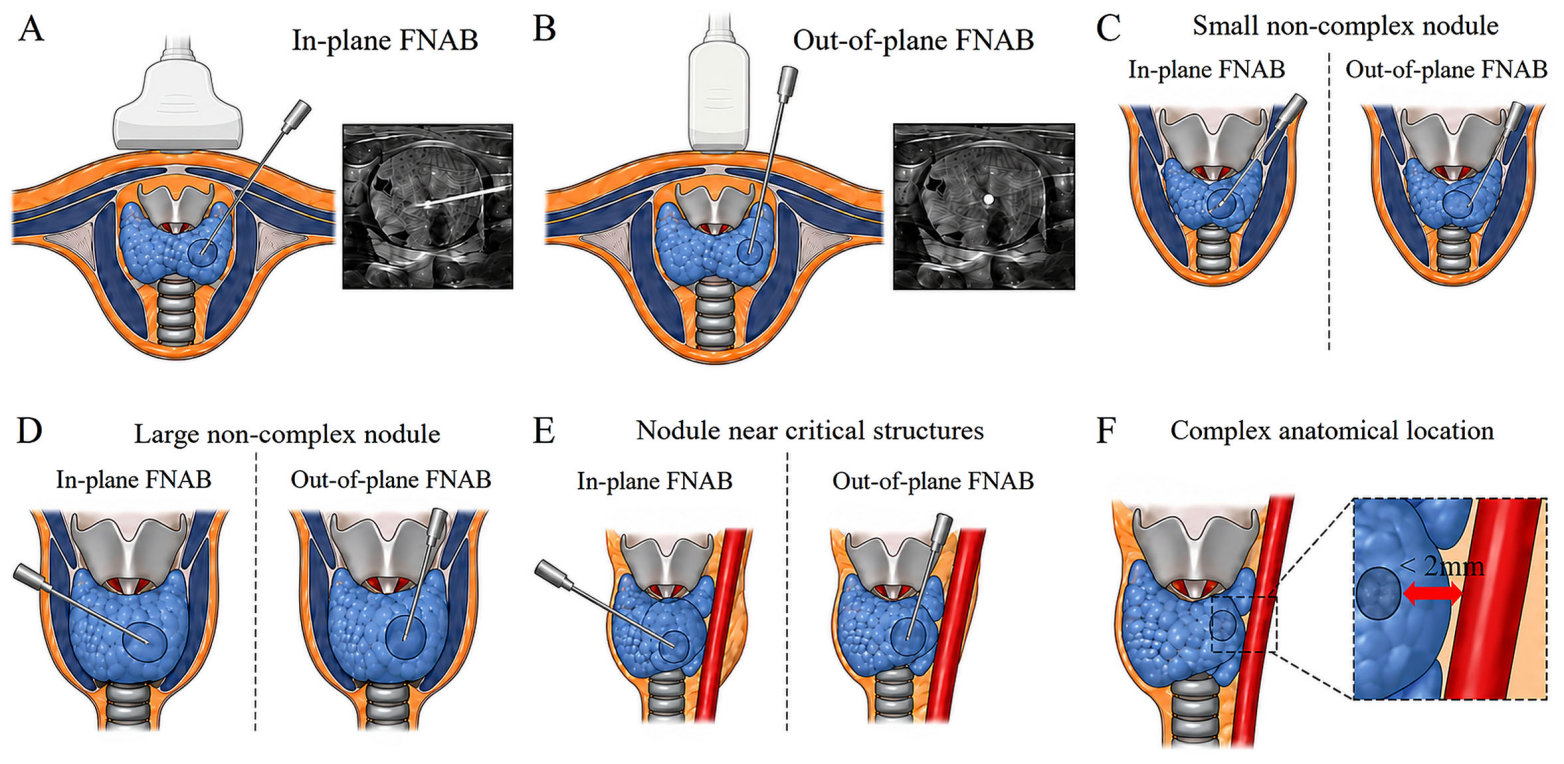
Conceptual schematic of in-plane and out-of-plane ultrasound-guided FNAB approaches and risk-adapted trajectory selection for thyroid nodules. **(A)** In-plane technique, in which the needle shaft and tip remain within the ultrasound imaging plane, allowing continuous visualization during target sampling. **(B)** Out-of-plane technique, in which the needle intersects the ultrasound imaging plane and the tip appears as a discrete echogenic dot with limited shaft visualization. **(C)** Small non-complex nodules, for which in-plane access may facilitate needle-tip confirmation and precise targeting. **(D)** Large non-complex nodules, for which either approach may be feasible depending on target accessibility, safe access route, and operator proficiency. **(E)** Complex anatomical scenarios adjacent to critical structures, in which trajectory selection should prioritize procedural safety and real-time needle-tip confirmation. **(F)** Complex anatomical location was defined as a nodule located within 2 mm of the trachea or a major cervical vessel. FNAB, fine-needle aspiration biopsy. Representative clinical ultrasound examples are provided in **Supplementary Figure 2**.

Approach selection in clinical practice was based on lesion accessibility, anticipated needle-tip visualization, and the planned safe access route. Procedure-related hematoma, the predefined safety endpoint, was defined as any hematoma detected on post-procedural ultrasound or documented in the procedural record. **Figure 2** illustrates the in-plane and out-of-plane approaches, risk-adapted trajectory selection, and the definition of complex anatomical location.

## Outcomes

Diagnostic adequacy was the primary outcome and was defined as Bethesda category II-VI cytology according to the Bethesda System for Reporting Thyroid Cytopathology (1). Bethesda category I cytology within the final analyzable cohort was considered nondiagnostic and was included in the analysis rather than excluded. Secondary descriptive cytological outcomes included nondiagnostic cytology and Bethesda V-VI cytology. Procedure-related hematoma was evaluated as the predefined safety outcome. Bethesda V-VI cytology was analyzed only as a cytological distribution category and was not interpreted as evidence of malignancy detection or final histopathological accuracy.

## Cytological assessment

Cytological interpretation was performed according to the Bethesda System for Reporting Thyroid Cytopathology at each participating center. All centers used the same Bethesda-based definition of diagnostic adequacy for study classification. Cytology results were extracted from formal institutional cytopathology reports. When necessary, Bethesda categories were centrally checked for coding consistency before analysis; however, centralized cytopathological slide review or repeated slide reading was not performed. Targeted re-review of all Bethesda I slides was also not feasible because original cytology slides were not uniformly retrievable across the participating centers under the retrospective anonymized data-authorization framework. Therefore, potential inter-laboratory variation in cytopathological interpretation, particularly for Bethesda I nondiagnostic specimens, could not be completely eliminated.

### Statistical analysis

Statistical analyses were performed using IBM SPSS Statistics version 27.0 (IBM Corp., Armonk, NY, USA). Propensity score-weighted analyses and Firth penalized logistic regression sensitivity analyses were conducted using R software, version 4.3.1 (R Foundation for Statistical Computing, Vienna, Austria). Continuous variables were assessed for normality using the Shapiro–Wilk test and are presented as medians with interquartile ranges; between-group comparisons were performed using the Mann–Whitney *U* test. Categorical variables are presented as counts and percentages and were compared using the chi-square test or Fisher’s exact test, as appropriate.

Multivariable logistic regression was used to evaluate the association between needle approach and diagnostic adequacy, with results expressed as odds ratios (ORs) and 95% confidence intervals (CIs). The primary model included needle approach, nodule size category, complex anatomical location, and operator experience. The same covariate structure was applied separately in the primary and external validation cohorts. In the pooled model, center source was additionally included, and an approach-by-cohort interaction term was used to assess between-cohort heterogeneity. An additional pooled model further adjusted for skin-to-nodule distance.

To address potential confounding by indication, stabilized inverse probability of treatment weighting (IPTW) was performed as a sensitivity analysis. The propensity score for receiving the in-plane approach was estimated using age, sex, maximum nodule diameter, nodule size category, complex anatomical location, skin-to-nodule distance, American College of Radiology Thyroid Imaging Reporting and Data System (ACR TI-RADS) category, suspicious cervical lymph node, operator experience, and center source. Covariate balance was assessed using absolute standardized mean differences, with values < 0.10 indicating acceptable balance. IPTW-weighted logistic regression models with robust standard errors were fitted.

Given the small number of nondiagnostic events, Firth penalized logistic regression was additionally performed using the pooled center-adjusted covariate structure. Needle-tip visualization was not included in the primary multivariable model because it was analyzed as a procedural process variable. Procedure-related hematoma was compared between approach groups as the predefined safety endpoint, and risk differences were calculated as descriptive effect measures when appropriate. All tests were two-sided, and *P* values < 0.05 were considered statistically significant. Subgroup analyses were exploratory; subgroup-specific ORs and 95% CIs were presented as descriptive estimates, and formal inference focused on tests for interaction rather than within-subgroup significance testing.

## Results

### Study population and cohort structure

The final analysis included 506 thyroid nodules, comprising 303 nodules in the primary cohort and 203 nodules in the external validation cohort. The external validation cohort included 113 nodules from the Union Hospital Affiliated to Fujian Medical University and 90 nodules from the Second Affiliated Hospital of Fujian Medical University. Overall, 289 nodules were sampled using the in-plane approach and 217 using the out-of-plane approach. The proportion of in-plane procedures did not differ significantly between the primary and external validation cohorts [177/303 (58.4%) vs. 112/203 (55.2%); *P* = 0.470; **Table 1**]. Basic patient demographics, including age and sex, are reported in **Tables 1** and **2**. No statistically significant between-cohort differences were observed in baseline characteristics, diagnostic adequacy, or procedure-related hematoma (all *P* > 0.05; **Table 1**).

**Table 1:** Cohort composition, baseline characteristics, and key outcomes by cohort.

Variable	Primary cohort (n = 303)	External validation cohort (n = 203)	P value
Needle approach, *n* (%)			0.470
In-plane	177 (58.4)	112 (55.2)	
Out-of-plane	126 (41.6)	91 (44.8)	
Age, years	52.0 (43.0–59.0)	51.0 (43.0–58.0)	0.536
Female sex, n (%)	225 (74.3)	161 (79.3)	0.190
Maximum diameter, cm	0.7 (0.5–2.0)	0.8 (0.4–1.7)	0.421
Nodule size, *n* (%)			0.984
<0.5 cm	85 (28.1)	56 (27.6)	
0.5–1.0 cm	101 (33.3)	67 (33.0)	
>1.0 cm	117 (38.6)	80 (39.4)	
Nodule ≤1.0 cm, n (%)	186 (61.4)	123 (60.6)	0.857
Complex anatomical location, n (%)	64 (21.1)	41 (20.2)	0.801
Skin-to-nodule distance, mm	13.5 (10.8–16.0)	13.6 (10.6–16.1)	0.608
ACR TI-RADS category, *n* (%)			0.605
TR3	16 (5.3)	7 (3.4)	
TR4	179 (59.1)	120 (59.1)	
TR5	108 (35.6)	76 (37.4)	
Senior operator, n (%)	201 (66.3)	130 (64.0)	0.594
Diagnostic adequacy, n (%)	281 (92.7)	189 (93.1)	0.876
Procedure-related hematoma, n (%)	22 (7.3)	16 (7.9)	0.864

Data are presented as median (interquartile range) or *n* (%). Between-cohort comparisons were performed using the Mann–Whitney *U* test, chi-square test, or Fisher’s exact test, as appropriate. Diagnostic adequacy was defined as Bethesda category II–VI cytology. Complex anatomical location was defined as a nodule located within 2 mm of the trachea or a major cervical vessel. ACR TI-RADS, American College of Radiology Thyroid Imaging Reporting and Data System.

**Table 2:** Baseline characteristics of the overall cohort according to needle approach.

Variable	In-plane (n = 289)	Out-of-plane (n = 217)	P value
Age, years	52.0 (45.0–59.0)	51.0 (41.0–58.0)	0.132
Female sex, n (%)	215 (74.4)	171 (78.8)	0.249
Maximum diameter, cm	0.6 (0.4–0.9)	1.6 (0.7–2.6)	<0.001
Nodule size, *n* (%)			<0.001
<0.5 cm	112 (38.8)	29 (13.4)	
0.5–1.0 cm	114 (39.4)	54 (24.9)	
>1.0 cm	63 (21.8)	134 (61.8)	
Nodule ≤1.0 cm, n (%)	226 (78.2)	83 (38.2)	<0.001
Complex anatomical location, n (%)	67 (23.2)	38 (17.5)	0.119
Skin-to-nodule distance, mm	13.7 (10.7–15.9)	13.5 (10.8–16.2)	0.696
ACR TI-RADS category, *n* (%)			0.064
TR3	14 (4.8)	9 (4.1)	
TR4	158 (54.7)	141 (65.0)	
TR5	117 (40.5)	67 (30.9)	
Senior operator, n (%)	182 (63.0)	149 (68.7)	0.183
Suspicious cervical lymph node, n (%)	15 (5.2)	6 (2.8)	0.176

Data are presented as median (interquartile range) or *n* (%). Between-group comparisons were performed using the Mann–Whitney *U* test, chi-square test, or Fisher’s exact test, as appropriate. Complex anatomical location was defined as a nodule located within 2 mm of the trachea or a major cervical vessel. ACR TI-RADS categories were analyzed as TR3, TR4, and TR5; TR4 subcategories were not analyzed separately.

### Baseline characteristics according to needle approach

Baseline characteristics of the overall cohort according to needle approach are summarized in **Table 2**. Nodules sampled using the in-plane approach had a smaller median maximum diameter than those sampled using the out-of-plane approach [0.6 cm (0.4–0.9) vs. 1.6 cm (0.7–2.6); P < 0.001]. Accordingly, nodules ≤ 1.0 cm were more frequent in the in-plane group than in the out-of-plane group (78.2% vs. 38.2%), whereas nodules >1.0 cm were more common in the out-of-plane group (61.8% vs. 21.8%; *P* < 0.001). This imbalance reflected real-world approach selection and potential confounding by indication rather than randomized allocation. It was therefore addressed by multivariable adjustment for nodule size category, sensitivity analyses using stabilized inverse probability of treatment weighting, and exploratory subgroup analyses by nodule size. Complex anatomical location was numerically more frequent in the in-plane group, although the difference was not statistically significant (23.2% vs. 17.5%; *P* = 0.119). Skin-to-nodule distance and other baseline variables, including age, sex, ACR TI-RADS category, operator experience, and suspicious cervical lymph node, did not differ significantly between the two approach groups. Operator-level descriptive information is provided in **Supplementary Table 1**. Overall, nine operators across the three participating centers contributed cases to the study; the median operator-level case volume was 40 cases (range, 22–114).

### Diagnostic, procedural, and safety outcomes

Diagnostic, procedural, and safety outcomes are summarized in **Table 3**. In the overall cohort, diagnostic adequacy was higher with the in-plane approach than with the out-of-plane approach (98.3% vs. 85.7%; absolute difference, 12.6 percentage points; *P* < 0.001). Bethesda I nondiagnostic cytology was less frequent in the in-plane group (1.7% vs. 14.3%; *P* < 0.001). The proportion of Bethesda V–VI cytology was also higher in the in-plane group than in the out-of-plane group (48.8% vs. 32.7%; *P* < 0.001), but this was reported only as a cytological distribution category and was not interpreted as evidence of improved malignancy detection. Satisfactory needle-tip visualization was more frequent with the in-plane approach (97.2% vs. 63.6%; *P* < 0.001). Documented immediate procedure-related hematoma occurred in 6.2% of in-plane procedures and 9.2% of out-of-plane procedures, with no statistically significant between-group difference (*P* = 0.234). The absolute difference in documented hematoma rate was 3.0 percentage points higher in the out-of-plane group. The occurrence of procedure-related hematoma and hematoma-size distribution among affected cases are shown in **Supplementary Figure 1**.

**Table 3:** Diagnostic, procedural, and safety outcomes by needle approach.

Outcome	In-plane (n = 289)	Out-of-plane (n = 217)	P value
Diagnostic adequacy, n (%)	284 (98.3)	186 (85.7)	<0.001
Bethesda I nondiagnostic cytology, n (%)	5 (1.7)	31 (14.3)	<0.001
Bethesda V–VI cytology, n (%)	141 (48.8)	71 (32.7)	<0.001
Satisfactory needle-tip visualization, n (%)	281 (97.2)	138 (63.6)	<0.001
Procedure-related hematoma, n (%)	18 (6.2)	20 (9.2)	0.234

Data are presented as *n* (%). Diagnostic adequacy was defined as Bethesda category II–VI cytology, and Bethesda category I was classified as nondiagnostic. Bethesda V–VI cytology was analyzed as a cytological distribution category and was not interpreted as final histopathological accuracy or improved malignancy detection. Procedure-related hematoma refers to immediate hematoma documented on post-procedural ultrasound or in the procedural record.

Primary cohort, external validation cohort, and pooled analyses.

The association between needle approach and diagnostic adequacy was evaluated separately in the primary and external validation cohorts and further examined in pooled models (**Table 4**). In the primary cohort, diagnostic adequacy was higher with the in-plane approach than with the out-of-plane approach [174/177 (98.3%) vs. 107/126 (84.9%)]. After adjustment for nodule size category, complex anatomical location, and operator experience, the in-plane approach remained significantly associated with diagnostic adequacy [adjusted odds ratio (OR), 9.39; 95% confidence interval (CI), 2.37–37.18; *P* = 0.001].

**Table 4:** Adjusted logistic regression analyses for diagnostic adequacy.

Model	Adjusted OR	95% CI	P value
Primary cohort	9.39	2.37–37.18	0.001
External validation cohort	14.51	2.82–74.74	0.001
Pooled center-adjusted model	12.10	4.28–34.20	<0.001
Pooled model additionally adjusted for skin-to-nodule distance	12.15	4.29–34.41	<0.001
Approach-by-cohort interaction	—	—	0.836

Multivariable logistic regression was used to evaluate the association between needle approach and diagnostic adequacy, with the out-of-plane approach as the reference. Primary and external validation cohort models were adjusted for nodule size category, complex anatomical location, and operator experience. Pooled models additionally adjusted for center source, and the final pooled model further adjusted for skin-to-nodule distance. The approach-by-cohort interaction tested whether the association differed between cohorts. OR, odds ratio; CI, confidence interval.

**Figure 3:**
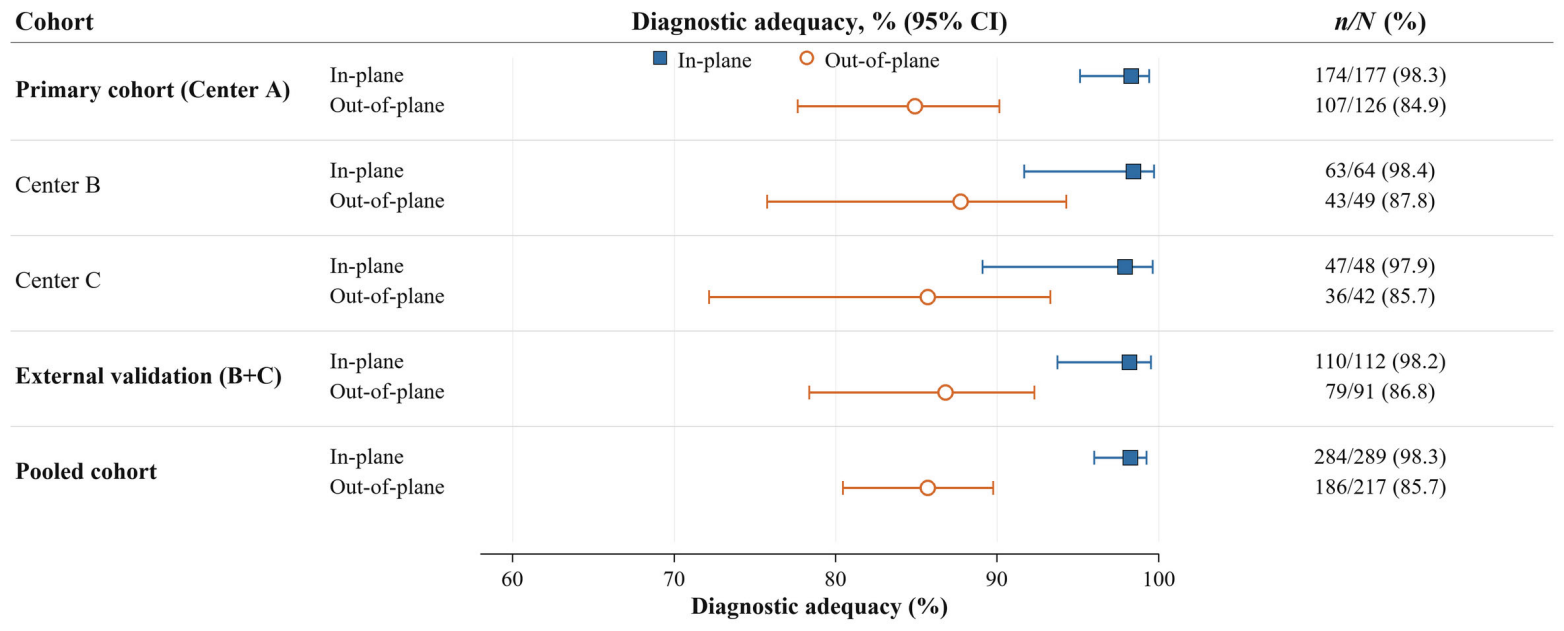
Diagnostic adequacy by study center and needle approach. Unadjusted diagnostic adequacy rates are shown for the in-plane and out-of-plane approaches in the primary cohort, each external validation center, the combined external validation cohort, and the pooled cohort. Squares indicate the in-plane approach, and open circles indicate the out-of-plane approach. Horizontal error bars indicate Wilson 95% confidence intervals. Diagnostic adequacy was defined as Bethesda category II–VI cytology.

The external validation cohort showed the same direction of association, with higher diagnostic adequacy after in-plane sampling [110/112 (98.2%) vs. 79/91 (86.8%)]. This association remained significant after applying the same covariate structure (adjusted OR, 14.51; 95% CI, 2.82–74.74; *P* = 0.001). Center-level estimates were directionally consistent across the primary center and both external validation centers: Center A [174/177 (98.3%) vs. 107/126 (84.9%)], Center B [63/64 (98.4%) vs. 43/49 (87.8%)], and Center C [47/48 (97.9%) vs. 36/42 (85.7%); **Figure 3**].

In the pooled center-adjusted model, the in-plane approach remained significantly associated with diagnostic adequacy (adjusted OR, 12.10; 95% CI, 4.28–34.20; *P* < 0.001). The result was essentially unchanged after additional adjustment for skin-to-nodule distance (adjusted OR, 12.15; 95% CI, 4.29–34.41; *P* < 0.001). The approach-by-cohort interaction was not statistically significant (*P* = 0.836), indicating no evidence that the association differed between the primary and external validation cohorts.

### Sensitivity analyses

In the stabilized inverse probability of treatment weighting (IPTW) sensitivity analysis, measured covariate balance improved substantially after weighting. The maximum absolute standardized mean difference decreased from 0.886 before weighting to 0.058 after weighting, and all post-weighting standardized mean differences were < 0.10 (**Supplementary Table 2**). The association between the in-plane approach and diagnostic adequacy remained significant after IPTW weighting (OR, 5.47; 95% CI, 1.44–20.79; *P* = 0.013), with similar findings in the IPTW-weighted covariate-adjusted model (OR, 5.57; 95% CI, 1.43–21.68; *P* = 0.013). In the additional Firth penalized logistic regression sensitivity analysis, the association also remained significant (OR, 10.77; 95% CI, 4.10–28.27; *P* < 0.001; **Supplementary Table 3**).

### Subgroup analysis

**Figure 4:**
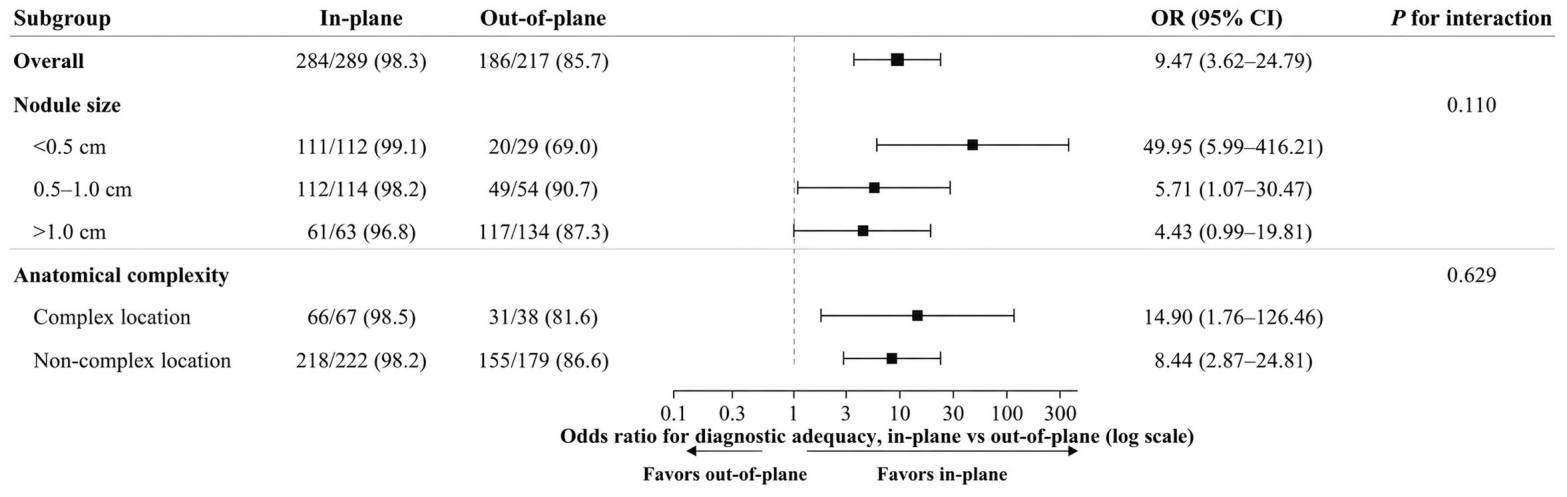
Exploratory subgroup analysis of diagnostic adequacy according to nodule size and anatomical complexity. Unadjusted odds ratios compare the in-plane approach with the out-of-plane approach, with the out-of-plane approach as the reference category. Squares indicate point estimates, and horizontal lines indicate 95% confidence intervals. Odds ratios and 95% confidence intervals were estimated using the log odds-ratio method. P values for interaction were calculated using likelihood-ratio tests comparing logistic regression models with and without approach-by-subgroup interaction terms. Subgroup analyses were exploratory, and several estimates had wide confidence intervals.

Exploratory subgroup analyses by nodule size and anatomical complexity are shown in **Figure 4**, with descriptive estimates provided in **Supplementary Table 4**. The direction of association generally favored the in-plane approach across predefined subgroups. However, several strata contained few nondiagnostic events, resulting in imprecise estimates and wide confidence intervals. Therefore, these results should not be interpreted as confirmatory evidence of subgroup-specific superiority. Tests for interaction did not show statistically significant heterogeneity by nodule size (*P* for interaction = 0.110) or anatomical complexity (*P* for interaction = 0.629). These findings should be interpreted as exploratory and hypothesis-generating.

### Needle-tip visualization process analysis

Needle-tip visualization was analyzed as a procedural process variable rather than as a baseline covariate. Satisfactory needle-tip visualization was more frequent with the in-plane approach than with the out-of-plane approach [281/289 (97.2%) vs. 138/217 (63.6%); *P* < 0.001; **Table 3**]. Interobserver agreement for needle-tip visualization assessment was high, with a Cohen’s κ of 0.82. In a descriptive process-level analysis, diagnostic adequacy was higher among nodules with satisfactory needle-tip visualization than among those with unsatisfactory visualization [413/419 (98.6%) vs. 57/87 (65.5%); *P* < 0.001; **Supplementary Table 5**]. These findings were descriptive and were not intended to establish a formal mediation effect or causal pathway.

## Discussion

In this multicenter retrospective cohort study, the in-plane approach was associated with higher diagnostic adequacy than the out-of-plane approach for ultrasound-guided FNAB of thyroid nodules. This association was consistent across the primary cohort and two external validation centers and remained significant after multivariable adjustment and sensitivity analyses. Satisfactory needle-tip visualization was more frequent with the in-plane approach and was associated with higher diagnostic adequacy in descriptive process-level analyses. The higher diagnostic adequacy observed with the in-plane approach was not accompanied by a statistically significant increase in immediate documented hematoma. Because this was a retrospective, nonrandomized study, these findings should be interpreted as preliminary and hypothesis-generating rather than as proof of causal superiority.

The difference in nondiagnostic cytology between groups provides the main clinical context for the observed association. Bethesda category I nondiagnostic cytology occurred in 1.7% of nodules sampled with the in-plane approach and 14.3% of those sampled with the out-of-plane approach (*P* < 0.001). Nondiagnostic cytology remains a persistent limitation of thyroid FNAB because it may lead to repeat biopsy, additional diagnostic procedures, prolonged surveillance, or delayed clinical decision-making (1). Previous studies have shown that nondiagnostic thyroid FNAB is multifactorial (12). Follow-up studies have reported the clinical course and timing considerations after nondiagnostic or indeterminate cytology (13–15). Studies evaluating repeat FNAB and core-needle biopsy after inconclusive cytology further support the need to minimize nondiagnostic sampling at the initial procedure (16–19). In addition, sonographic characteristics and strict guideline implementation have been associated with nondiagnostic or inconclusive cytology results (20, 21). In the present study, needle approach and needle-tip confirmation represent potentially modifiable procedural factors, but their apparent association with adequacy should be interpreted alongside the nonrandomized distribution of nodule size and other case-mix variables.

A similar direction of effect was observed in the primary cohort and in both external validation centers, and the approach-by-cohort interaction was not statistically significant. The association also remained significant after center adjustment, stabilized IPTW weighting, and Firth penalized logistic regression. These analyses support the consistency of the observed association across clinical settings, although residual center-level, operator-level, lesion-level, and workflow-related confounding cannot be fully excluded.

The association between the in-plane approach and diagnostic adequacy is procedurally plausible but should not be interpreted as evidence of a formal mediation effect. Smaller thyroid nodules may be more vulnerable to off-target sampling because minor deviations in needle-tip position can move the sampling site outside the intended target. In the present cohort, nodules sampled with the in-plane approach were smaller than those sampled with the out-of-plane approach, and nodules ≤ 1.0 cm accounted for 78.2% of lesions in the in-plane group. This pattern is consistent with the clinical need for reliable needle-tip confirmation in small targets. However, needle-tip visualization was not randomized and may be influenced by acoustic window quality, lesion accessibility, operator judgment, and documentation fidelity; therefore, it should be interpreted as supportive procedural evidence rather than as a validated mediator.

The clinical implication of this study lies in refining a risk-adapted trajectory-selection concept rather than validating a fixed procedural algorithm. Real-world thyroid FNAB approach selection is influenced by target size, proximity to critical structures, safe access corridor, operator proficiency, and expected needle-tip confirmation. Small nodules may favor an in-plane trajectory when continuous needle-tip visualization is technically feasible, whereas either approach may be reasonable for larger, anatomically uncomplicated nodules if adequate target access and tip confirmation can be achieved. For nodules adjacent to the trachea or major cervical vessels, trajectory selection should prioritize procedural safety, including consideration of an out-of-plane trajectory when it provides a shorter or safer access route. These considerations require prospective validation before clinical algorithmic use.

Although thyroid FNAB is generally considered a low-risk procedure, safety remains important when comparing technical approaches (11). We selected immediate documented hematoma as the predefined safety endpoint because it could be ascertained from post-procedural ultrasound assessment or procedural documentation. Previous studies of thyroid biopsy techniques have evaluated hematoma, pain, tolerability, vasovagal reactions, and other complications as relevant safety outcomes (22–24). In the present cohort, the immediate hematoma rate was 6.2% in the in-plane group and 9.2% in the out-of-plane group, with no statistically significant between-group difference (*P* = 0.234). The observed hematoma rates may partly reflect post-procedural ultrasound assessment, which can identify small, self-limiting hematomas that may not be detected during symptom-driven follow-up alone. Notably, pain scores, vasovagal reactions, delayed adverse events, and other patient-reported outcomes were not systematically collected. Therefore, these safety findings should be interpreted as showing no apparent excess risk of immediate hematoma with the in-plane approach, rather than as evidence of superior overall procedural safety.

Several limitations should be acknowledged. First, this was a retrospective, nonrandomized study, and needle approach was selected during routine clinical practice. Although multivariable adjustment, center adjustment, propensity score-weighted sensitivity analyses, and Firth penalized regression were performed, residual confounding, complete-case selection bias, and case-mix differences cannot be excluded. The imbalance in nodule size between approach groups is an important example of potential confounding by indication. Second, 41 screened nodules lacked retrievable formal Bethesda cytology reports and were excluded before outcome analysis. These cases were distinct from Bethesda I nondiagnostic cases, which were retained in the final analysis, but the specific reasons for unavailable cytology could not be reliably reconstructed from anonymized retrospective records. Third, diagnostic adequacy was based on institutional Bethesda cytology reports. Centralized cytopathological slide review or repeated reading was not performed, and targeted re-review of Bethesda I slides was not feasible because original cytology slides were not uniformly retrievable across centers. Therefore, inter-laboratory variation in Bethesda classification could not be fully assessed, and the findings should not be interpreted as evidence of improved malignancy detection or final histopathological accuracy. Fourth, detailed lesion-level characteristics such as fibrosis, coarse or rim calcification, and other factors affecting needle passage or specimen adequacy were not consistently recorded across centers and were not included as separate covariates. Finally, needle-tip visualization was assessed retrospectively from archived ultrasound materials. Reviewers could not be fully blinded to needle approach, dynamic video availability was not prospectively standardized, and static images may be less reliable for confirming needle-tip position during sampling. Operator experience was also summarized using a simplified senior/non-senior classification, which could not fully capture individual proficiency, preference, learning-curve effects, or operator-level clustering.

Future prospective studies should standardize dynamic video acquisition, needle-tip visualization assessment, operator-level documentation, sampling technique, procedural difficulty, nodule calcification patterns, fibrosis-related features, cytopathological re-review, and final reference diagnoses when available. Predefined stratification by nodule size and anatomical complexity may help evaluate the generalizability and clinical utility of the proposed risk-adapted trajectory-selection framework. Standardized cytology-support workflows, such as ROSE, telecytology, or structured adequacy assessment, should also be considered (25).

In conclusion, the in-plane approach was associated with higher diagnostic adequacy than the out-of-plane approach in ultrasound-guided thyroid FNAB. This association showed a consistent direction across the primary cohort and two external validation centers and persisted in multivariable and sensitivity analyses. The higher diagnostic adequacy observed with the in-plane approach was not accompanied by a statistically significant increase in immediate documented hematoma. These findings provide preliminary, hypothesis-generating multicenter evidence that may inform risk-adapted trajectory selection considering nodule size, anatomical complexity, access safety, and expected needle-tip confirmation. Prospective validation is required before this concept can be used as a formal decision algorithm.

## Data Availability

The de-identified data supporting the findings of this study may be made available from the corresponding author upon reasonable request, subject to institutional approval and applicable data-sharing regulations.
