# Supplementary figures and images for: Risk-adapted trajectory selection for ultrasound-guided thyroid fine-needle aspiration biopsy: a multicenter study of in-plane and out-of-plane approaches
Source: Front Endocrinol (Lausanne). 2026 Jul 17;17:1908297. doi: 10.3389/fendo.2026.1908297 (PMC13423726; doi:10.3389/fendo.2026.1908297)

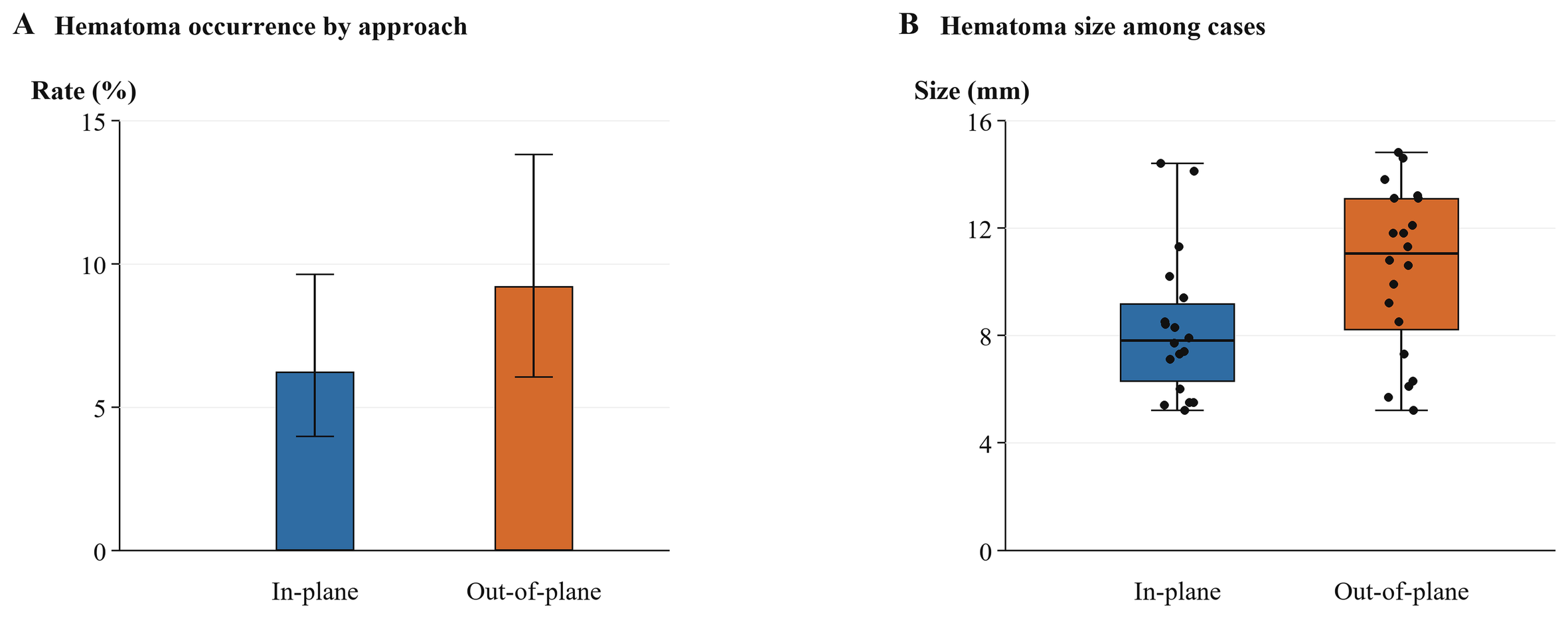

Supplement: Supplementary file 1 [file Image1.tif]

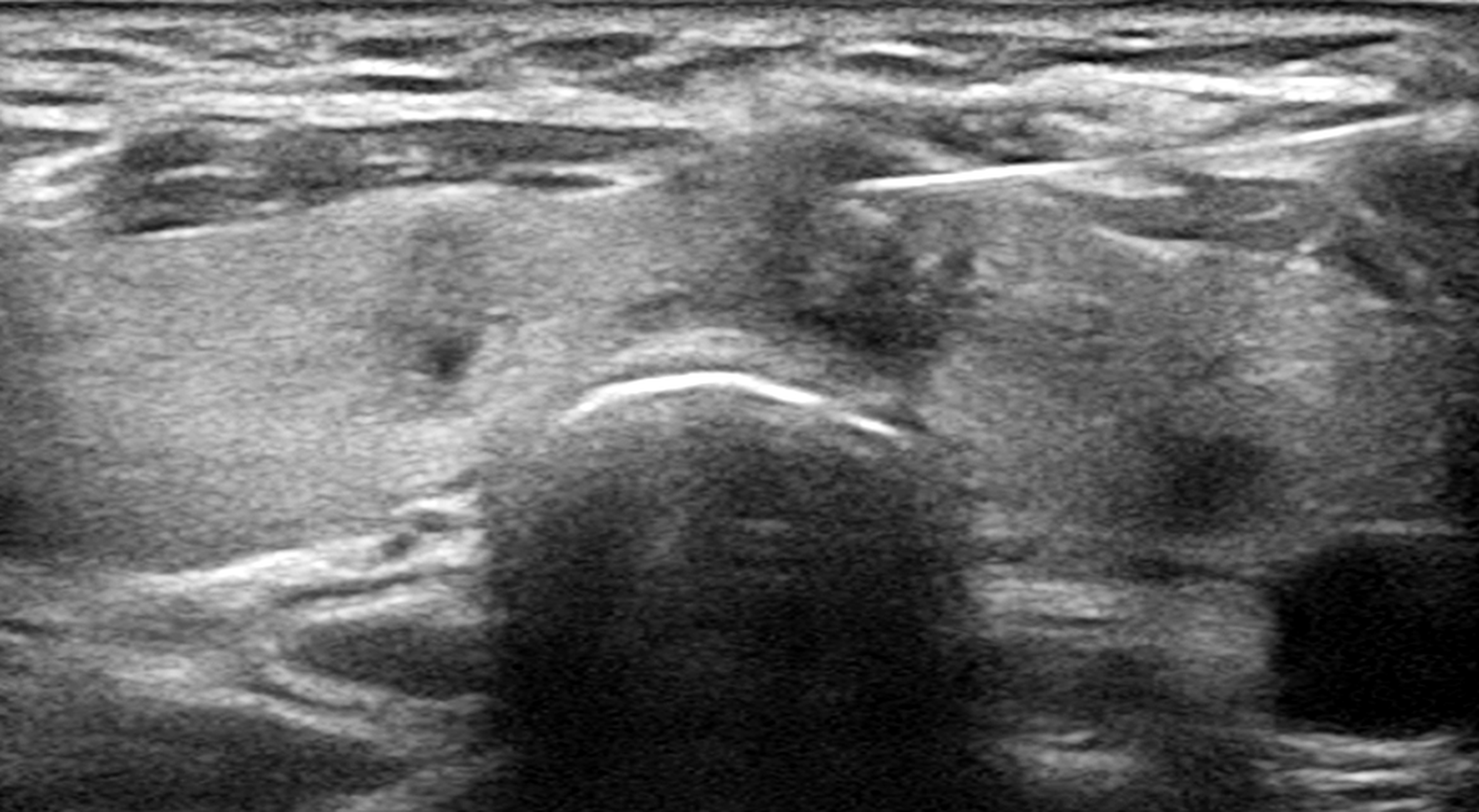

Supplement: Supplementary file 2 [file Image2.tif]

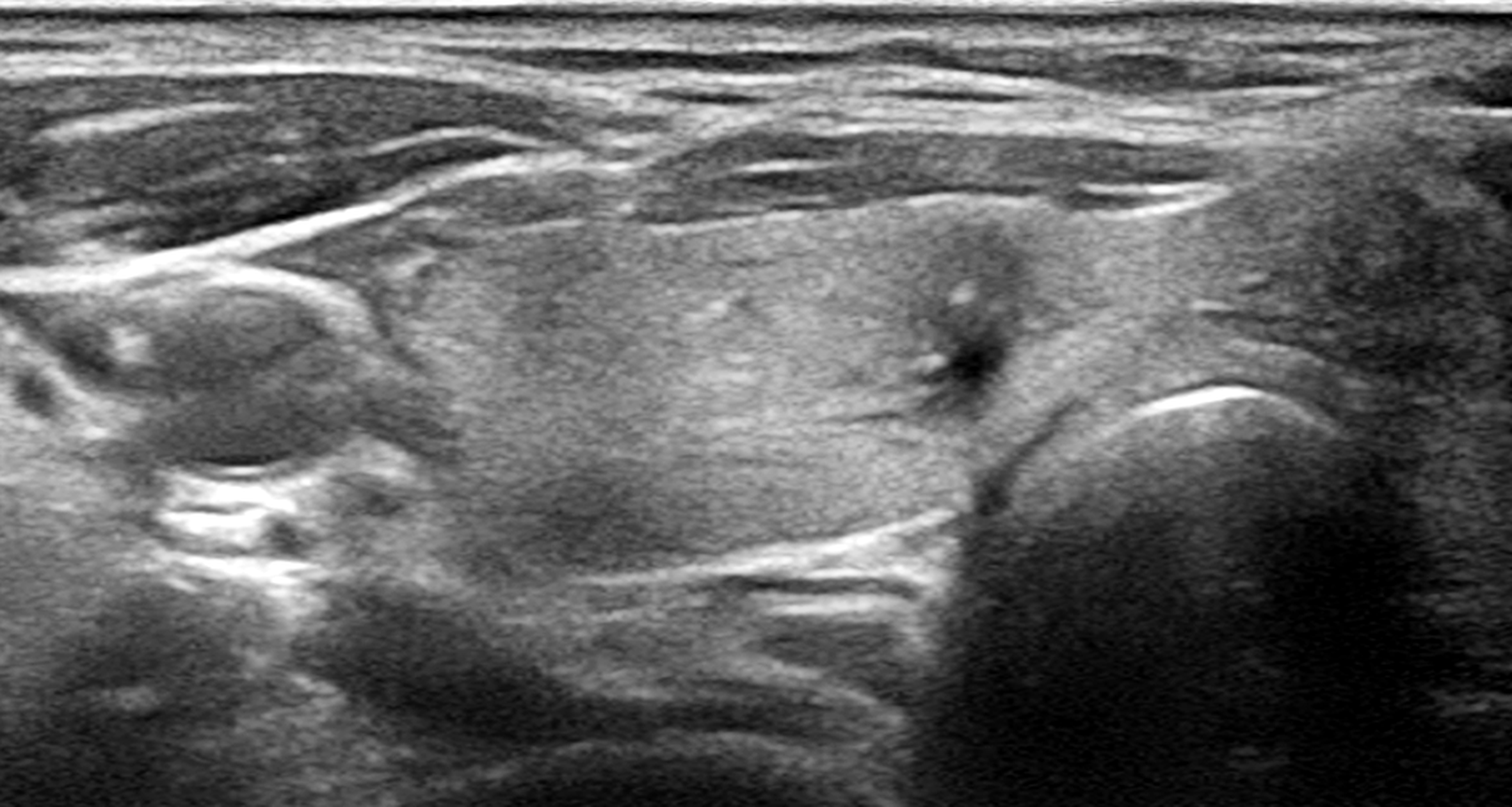

Supplement: Supplementary file 3 [file Image3.tif]

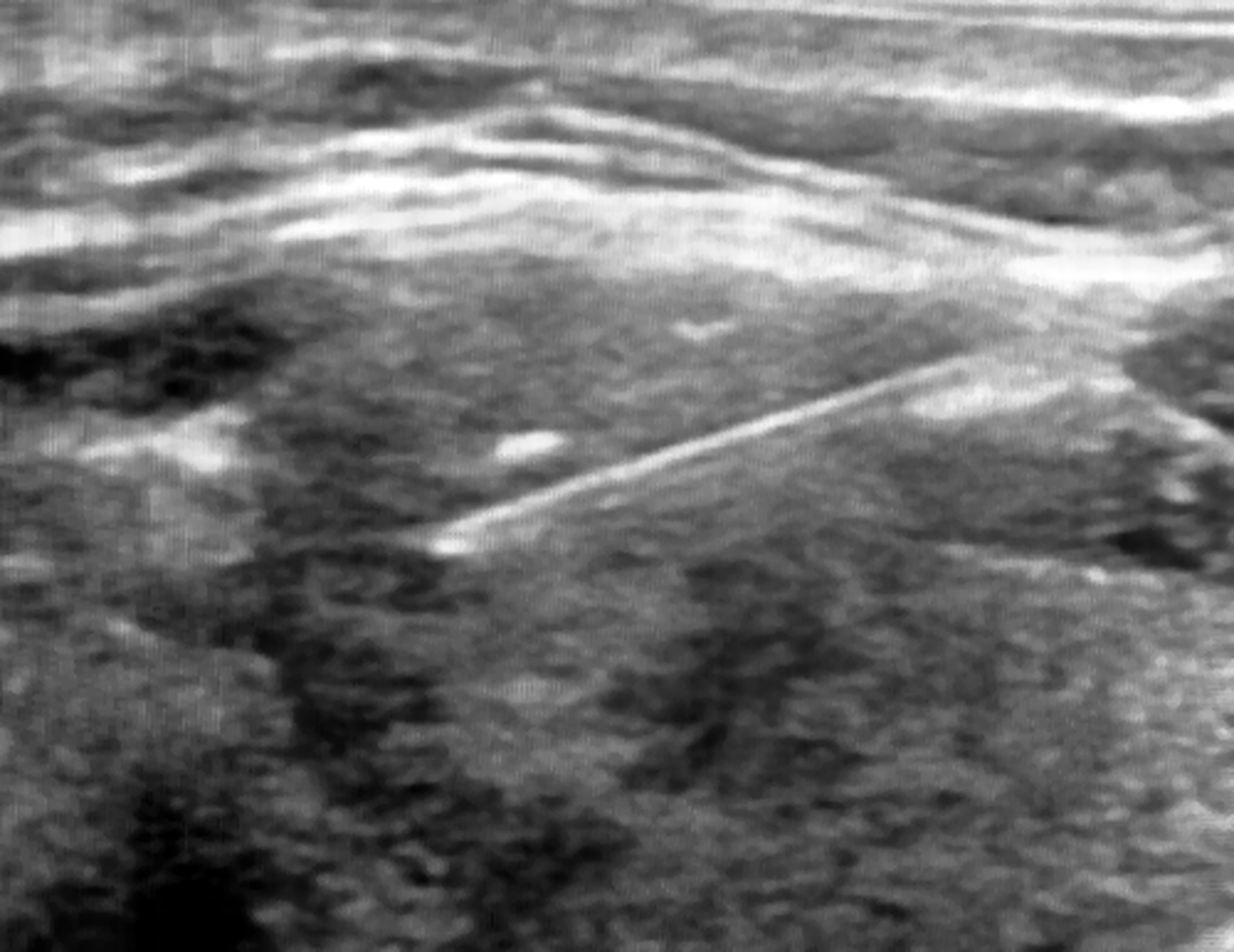

Supplement: Supplementary file 4 [file Image4.tif]

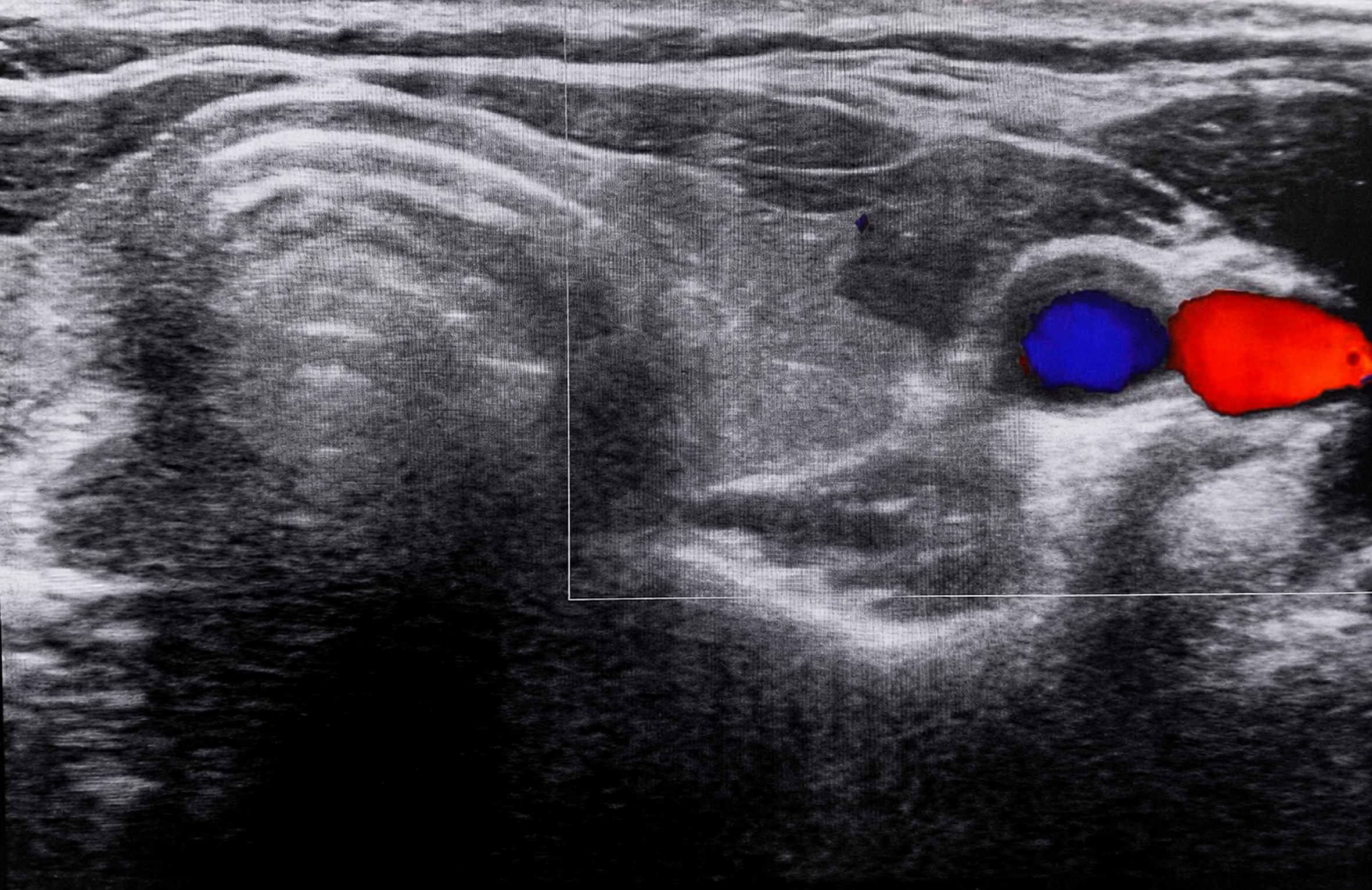

Supplement: Supplementary file 5 [file Image5.tif]

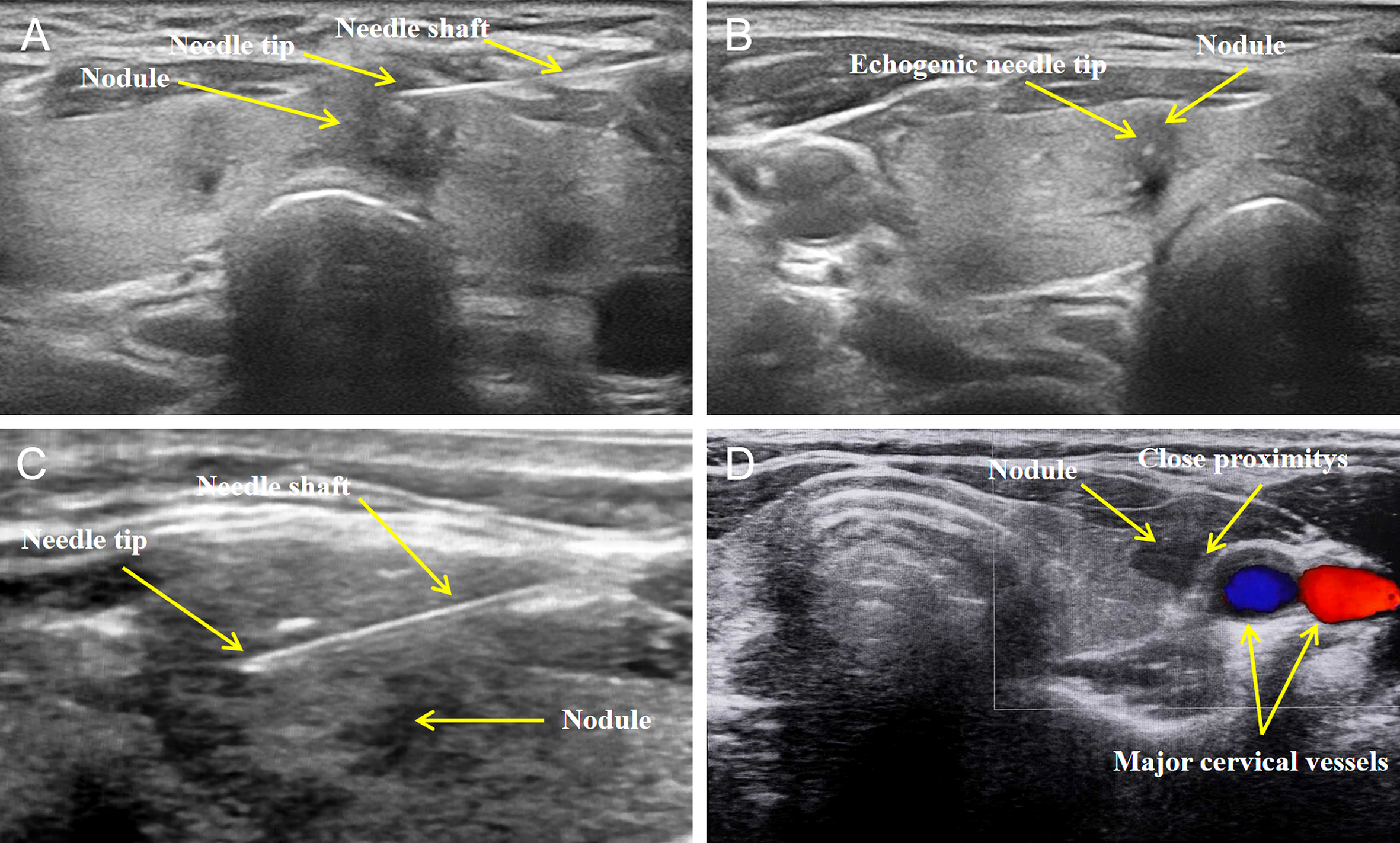

Supplement: Supplementary file 6 [file Image6.tif]
