# Supplementary material for: Risk-adapted trajectory selection for ultrasound-guided thyroid fine-needle aspiration biopsy: a multicenter study of in-plane and out-of-plane approaches
Source: Front Endocrinol (Lausanne). 2026 Jul 17;17:1908297. doi: 10.3389/fendo.2026.1908297 (PMC13423726; doi:10.3389/fendo.2026.1908297)
Supplement: Supplementary file 7 [file Table1.docx]

Supplementary **Figure S1**. Procedure-related hematoma according to needle approach. (A) Occurrence of procedure-related hematoma by needle approach. Bars indicate proportions, and error bars indicate Wilson 95% confidence intervals. (B) Hematoma size among cases with procedure-related hematoma. Box plots show medians and interquartile ranges; whiskers indicate observed ranges, and dots represent individual cases.

Supplementary **Figure S2**. Representative clinical ultrasound examples of ultrasound-guided thyroid FNAB. (A) In-plane FNAB showing the needle shaft and needle tip along the ultrasound imaging plane during target sampling. (B) Out-of-plane FNAB showing the needle tip as a focal echogenic point during target sampling. (C) Magnified example of needle-tip visualization, showing the needle tip relative to the target nodule. (D) Complex anatomical location on color Doppler ultrasound, showing a thyroid nodule in close proximity to major cervical vessels. Yellow arrows indicate the labeled structures in each panel. FNAB, fine-needle aspiration biopsy.

Supplementary Table S1. Operator-level descriptive information by cohort

| Variable | Primary cohort | External validation cohort | Overall |
| --- | --- | --- | --- |
| Number of center-specific operators | 3 | 6 | 9 |
| Senior operators, *n* (%) | 2 (66.7) | 4 (66.7) | 6 (66.7) |
| Median case volume per operator, *n* (range) | 102 (87–114) | 34 (22–46) | 40 (22–114) |
| Operators using both in-plane and out-of-plane approaches, *n* (%) | 3 (100.0) | 6 (100.0) | 9 (100.0) |
| Median proportion of in-plane procedures per operator, % (range) | 62.1 (48.2–66.7) | 55.9 (45.5–63.0) | 57.6 (45.5–66.7) |

Note: Operator identifiers were treated as center-specific anonymized operator codes. Senior operators were defined as attending-level or higher ultrasound physicians with at least 5 years of independent thyroid FNAB experience. Case volume refers to the number of included FNAB procedures performed by each center-specific operator in the study cohort. FNAB, fine-needle aspiration biopsy.

Supplementary Table S2. Covariate balance before and after stabilized IPTW

| Variable | SMD before IPTW | SMD after IPTW |
| --- | --- | --- |
| Age, years | 0.173 | 0.058 |
| Female sex | 0.104 | 0.002 |
| Maximum diameter, cm | 0.859 | 0.033 |
| Nodule size category | — | — |
| <0.5 cm | 0.604 | 0.004 |
| 0.5–1.0 cm | 0.316 | 0.004 |
| >1.0 cm | 0.886 | 0.00 |
| Complex anatomical location | 0.141 | 0.004 |
| Skin-to-nodule distance, mm | 0.042 | 0.008 |
| ACR TI-RADS category | — | — |
| TR3 | 0.034 | 0.028 |
| TR4 | 0.211 | 0.012 |
| TR5 | 0.202 | 0.00 |
| Suspicious cervical lymph node | 0.124 | 0.025 |
| Senior operator | 0.120 | 0.004 |
| Center source | — | — |
| Center A | 0.065 | 0.006 |
| Center B | 0.010 | 0.012 |
| Center C | 0.072 | 0.003 |

Note: Absolute standardized mean differences (SMDs) were used to assess covariate balance before and after stabilized inverse probability of treatment weighting (IPTW). The propensity score model included age, sex, maximum nodule diameter, nodule size category, complex anatomical location, skin-to-nodule distance, ACR TI-RADS category, suspicious cervical lymph node, operator experience, and center source. Maximum nodule diameter and nodule size category were both included to balance continuous size information and clinically predefined size strata. Values <0.10 were considered indicative of acceptable balance. The maximum absolute SMD decreased from 0.886 before IPTW to 0.058 after IPTW, and all post-IPTW absolute SMDs were <0.10. ACR TI-RADS, American College of Radiology Thyroid Imaging Reporting and Data System.

Supplementary Table S3. Sensitivity analyses for the association between needle approach and diagnostic adequacy

| Model | OR | 95% CI | *P* value |
| --- | --- | --- | --- |
| Unweighted crude logistic regression | 9.47 | 3.62–24.79 | <0.001 |
| Pooled center-adjusted multivariable logistic regression | 12.10 | 4.28–34.20 | <0.001 |
| IPTW-weighted marginal logistic regression | 5.47 | 1.44–20.79 | 0.013 |
| IPTW-weighted covariate-adjusted logistic regression | 5.57 | 1.43–21.68 | 0.013 |
| Firth penalized logistic regression | 10.77 | 4.10–28.27 | <0.001 |

Note: The out-of-plane approach was used as the reference. The pooled center-adjusted multivariable model was adjusted for nodule size category, complex anatomical location, operator experience, and center source. IPTW models used stabilized weights and robust standard errors. Firth penalized logistic regression was performed as an additional sensitivity analysis using the same covariate structure as the pooled center-adjusted model. CI, confidence interval; IPTW, inverse probability of treatment weighting; OR, odds ratio.

Supplementary Table S4. Subgroup analysis of diagnostic adequacy according to nodule size and anatomical complexity

| Subgroup | In-plane, n/N (%) | Out-of-plane, n/N (%) | OR (95% CI) |
| --- | --- | --- | --- |
| Overall | 284/289 (98.3) | 186/217 (85.7) | 9.47 (3.62–24.79) |
| Nodule size |  |  |  |
| <0.5 cm | 111/112 (99.1) | 20/29 (69.0) | 49.95 (5.99–416.21) |
| 0.5–1.0 cm | 112/114 (98.2) | 49/54 (90.7) | 5.71 (1.07–30.47) |
| >1.0 cm | 61/63 (96.8) | 117/134 (87.3) | 4.43 (0.99–19.81) |
| Anatomical complexity |  |  |  |
| Complex anatomical location | 66/67 (98.5) | 31/38 (81.6) | 14.90 (1.76–126.46) |
| Non-complex anatomical location | 218/222 (98.2) | 155/179 (86.6) | 8.44 (2.87–24.81) |

Note: Data are presented as the number of diagnostically adequate nodules/total nodules (%). Odds ratios (ORs) compare the in-plane approach with the out-of-plane approach, with the out-of-plane approach as the reference category. ORs and 95% confidence intervals (CIs) were estimated using the log odds-ratio method and are presented as descriptive estimates. Tests for interaction were calculated using likelihood-ratio tests comparing logistic regression models with and without approach-by-subgroup interaction terms. Subgroup analyses were exploratory and should not be interpreted as confirmatory evidence of subgroup-specific superiority. CI, confidence interval; OR, odds ratio.

Supplementary Table S5. Descriptive process-level analysis of needle-tip visualization and diagnostic adequacy

| Analysis/category | Favorable outcome, *n* (%) | Unfavorable outcome, *n* (%) | OR (95% CI) | *P* value |
| --- | --- | --- | --- | --- |
| Needle-tip visualization according to needle approach |  |  |  |  |
| In-plane | 281 (97.2) | 8 (2.8) | 20.11 (9.44–42.81) | <0.001 |
| Out-of-plane | 138 (63.6) | 79 (36.4) | 1.00 (reference) |  |
| Diagnostic adequacy according to needle-tip visualization |  |  |  |  |
| Satisfactory visualization | 413 (98.6) | 6 (1.4) | 36.23 (14.44–90.75) | <0.001 |
| Unsatisfactory visualization | 57 (65.5) | 30 (34.5) | 1.00 (reference) |  |

Note: Data are presented as *n* (%). In the first analysis, the favorable outcome was satisfactory needle-tip visualization; in the second analysis, the favorable outcome was diagnostic adequacy. Odds ratios (ORs) are crude estimates for descriptive process-level associations and should not be interpreted as causal effects. The out-of-plane approach and unsatisfactory needle-tip visualization were used as the reference categories, respectively. *P* values were calculated using the chi-square test or Fisher’s exact test, as appropriate. CI, confidence interval; OR, odds ratio.
